# Supplementary material for: CD151 expression marks atrial- and ventricular- differentiation from human induced pluripotent stem cells
Source: Commun Biol. 2024 Feb 28;7:231. doi: 10.1038/s42003-024-05809-2 (PMC10901864; doi:10.1038/s42003-024-05809-2)
Supplement: Supplementary file 2 — Supplementary Information [file 42003_2024_5809_MOESM2_ESM.pdf]

Supplementary information for

CD151 expression marks atrial- and ventricular- differentiation from human induced pluripotent stem cells

Supplementary Figures

Figure S1
Figure S2
Figure S3
Figure S4
Figure S5
Figure S6
Figure S7
Figure S8

Supplementary Tables

Supplementary Table 1
Supplementary Table 2
Supplementary Table 3

Figure S1

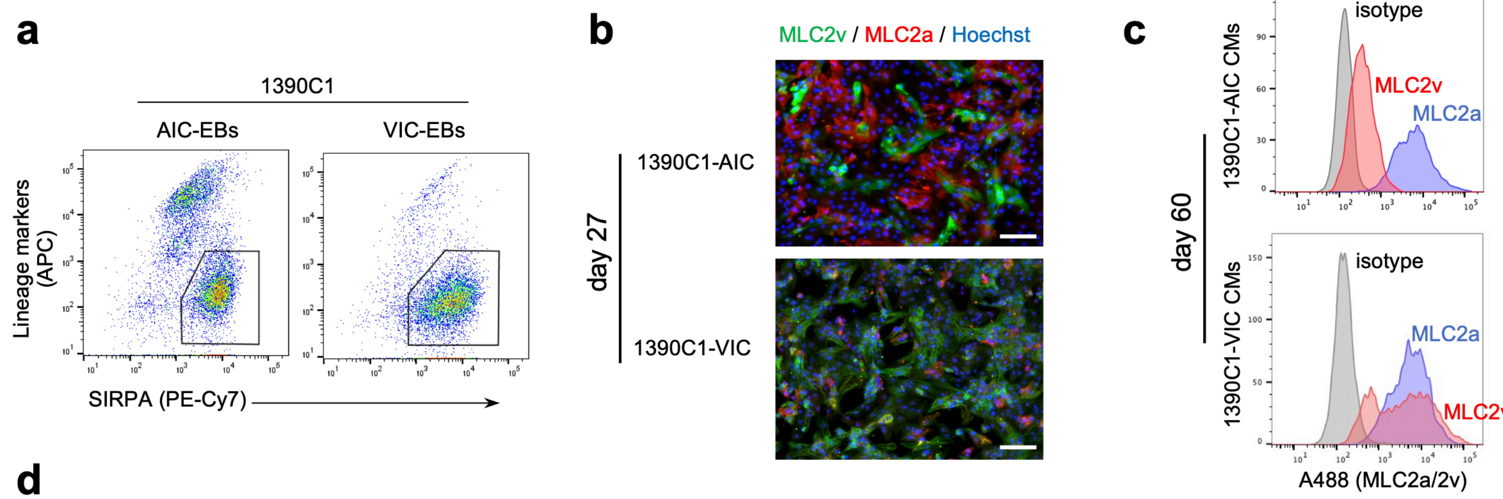

d

	VIC	AIC
control	1	1
CD1a	1	1
CD1b	1	1
CD1d	1	1
CD2	1	1
CD3	1	1
CD4	1	1
CD4v4	1	1
CD5	1	1
CD6	1	1
CD7	1	1
CD8a	1	1
CD8b	1	1
CD9	1	1
CD10	1	1
CD11a	1	1
CD11b	1	1
CD11c	1	1
CD13	1	1
CD14	1	1
CD15	1	1
CD15s	1	1
CD16	1	2
CD18	1	1
CD19	1	1
CD20	1	1
CD21	1	1
CD22	1	1
CD23	1	1
CD24	44	28
CD25	1	1

	VIC	AIC
CD26	1	1
CD27	1	1
CD28	2	2
CD29	89	87
CD30	3	1
CD31	1	1
CD32	1	1
CD33	1	1
CD34	1	1
CD35	1	1
CD36	1	2
CD37	1	1
CD38	1	2
CD39	1	3
CD40	1	5
CD41a	1	1
CD41b	1	1
CD42a	1	1
CD42b	1	1
CD43	40	27
CD44	39	45
CD45RA	1	2
CD45RB	2	1
CD45RO	3	4
CD45	1	1
CD46	84	76
CD47	87	67
CD48	1	1
CD49a	9	3
CD49b	1	6
CD49c	23	31

	VIC	AIC
CD49d	1	1
CD49e	5	3
CD49f	90	99
CD50	1	1
CD51-61	1	1
CD53	1	1
CD54	21	18
CD55	33	6
CD56	37	50
CD57	34	16
CD58	90	90
CD59	100	99
CD61	1	1
CD62E	1	1
CD62L	1	1
CD62P	1	1
CD63	5	4
CD64	1	1
CD66	2	1
CD66b	1	1
CD66f	1	1
CD69	3	1
CD70	1	1
CD71	44	21
CD72	1	1
CD73	1	1
CD74	1	1
CD75	1	1
CD77	1	1
CD79b	2	1
CD80	1	1

	VIC	AIC
CD81	11	4
CD83	2	1
CD84	4	1
CD85	2	1
CD86	1	0
CD87	1	0
CD88	1	0
CD89	1	0
CD90	2	0
CD91	1	0
CDw93	1	0
CD94	1	0
CD95	1	0
CD97	1	0
CD98	20	86
CD99	1	1
CD99R	1	0
CD100	1	0
CD102	1	1
CD103	1	0
CD104	1	10
CD105	1	0
CD106	1	0
CD107a	1	0
CD107b	1	0
CD108	1	0
CD109	1	0
CD112	1	0
CD114	0	1
CD116	1	0
CD117	1	0

	VIC	AIC
CD118	1	0
CD119	1	0
CD120a	1	0
CD120b	0	1
CD121a	1	0
CD121b	1	0
CD122	1	0
CD123	1	0
CD124	1	0
CD126	1	0
CD127	1	1
CD128b	1	2
CD130	1	4
CD132	1	1
CD134	1	1
CD135	1	0
CD137	1	0
CD137 Ligand	1	0
CD138	1	0
CD140a	1	1
CD140b	3	0
CD141	1	0
CD142	1	1
CD144	1	1
CD146	1	2
CD147	100	98
CD150	1	0
CD151	76	20
CD152	0	0
CD153	1	0
CD154	1	0

	VIC	AIC
CD158a	1	0
CD158b	1	0
CD161	1	0
CD162	1	0
CD163	1	1
CD164	28	21
CD165	3	5
CD166	33	3
CD171	1	0
CD172b	1	0
CD177	1	0
CD178	1	0
CD180	1	0
CD181	1	0
CD183	1	0
CD184	1	0
CD193	1	1
CD195	1	1
CD196	1	1
CD197	1	1
CD200	2	7
CD201	9	2
CD205	1	0
CD206	1	0
CD209	1	0
CD210	1	0
CD212	1	0
CD220	1	0
CD221	2	13
CD226	1	0
CD227	1	0

	VIC	AIC
CD229	1	1
CD231	1	0
CD235a	0	0
CD243	1	1
CD244	1	1
CD255	1	0
CD267	0	1
CD268	1	0
CD271	1	1
CD273	1	0
CD274	1	0
CD275	0	0
CD278	0	0
CD279	2	2
CD282	1	2
CD294	1	1
CD305	2	1
CD309	2	2
CD314	1	2
CD321	82	91
CDw327	1	2
CDw328	2	1
CD329	2	1
CD335	2	1
CD336	2	1
CD337	1	1
CD338	1	2
CD340	98	99
abTCR	1	2
b2microglobulin	17	7
BLTR-1	0	1

	VIC	AIC
CLIP	1	1
CMRF-44	2	2
CMRF-56	2	1
EGFR	3	37
fMLP-R	2	1
gdTCR	2	2
Hemprogenitorcell	2	2
HLA-ABC	23	18
HLA-A2	34	29
HLA-DQ	10	3
HLA-DR	2	2
HLA-DRDPDQ	2	1
invariantNKT	3	1
GD2	3	3
MICA-B	3	1
NKB1	2	2
SSEA-1	2	2
SSEA-3	7	1
SSEA-4	99	49
TRA1-60	1	1
TRA1-81	1	2
Vb23	2	2
Vb8	2	2
CD326	3	13
CLA	1	0
Integrinb7	1	1
mIgM	2	1
mIgG1	1	1
mIgG2a	3	1
mIgG2b	2	1
mIgG3	2	2

0
5
10
20
22
25
27
30
32
35
37
40
42
45
48
50
60
63
67
70
72
75
77
80
82
86
88
90
92
96
100

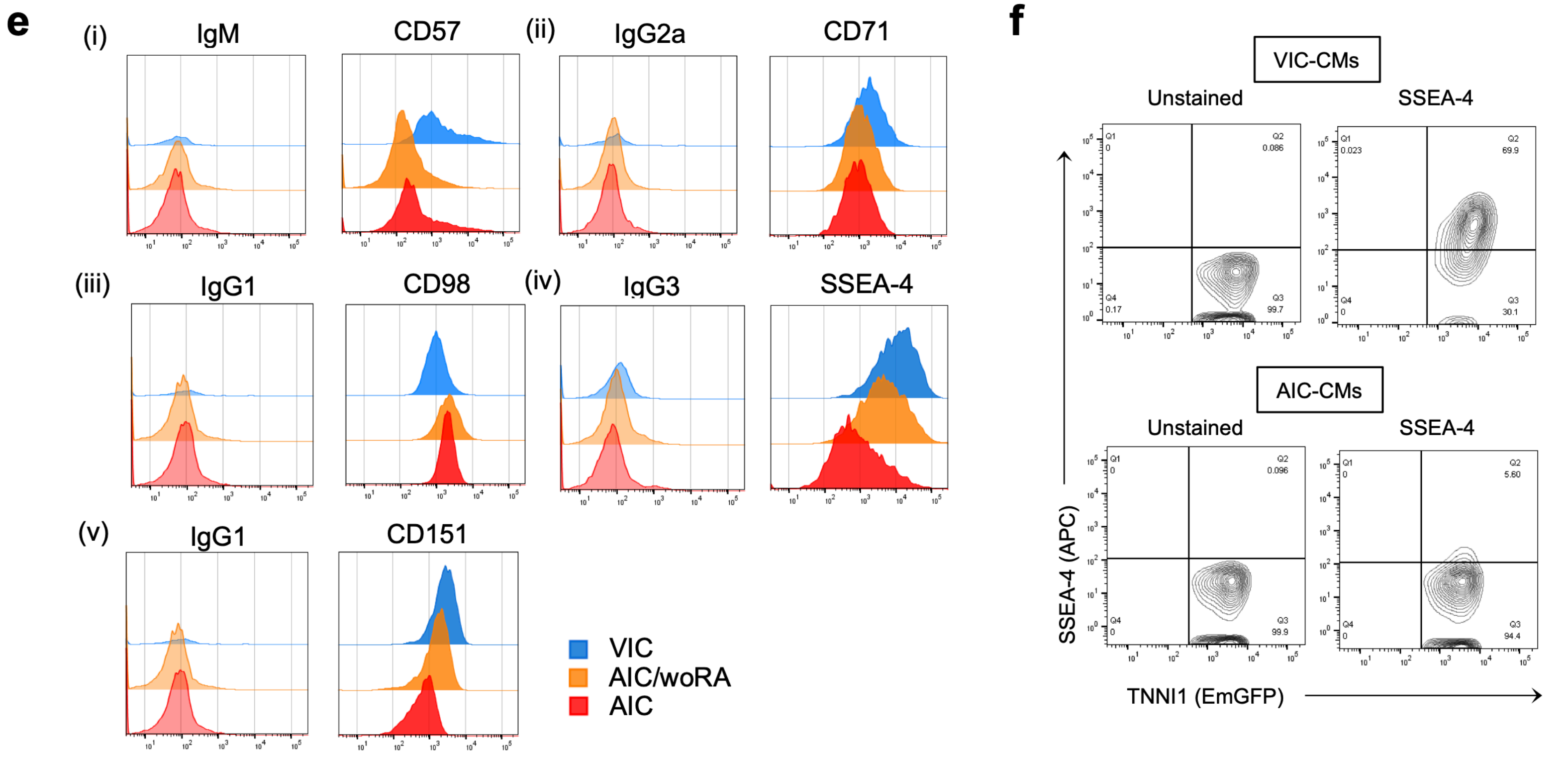

Figure S1. Cardiac differentiation in an independent hiPSC line and cell surface marker screening, related to Figure 1.

(a) Representative flow cytometry images of day 20 AIC- and VIC-EBs stained with SIRPA and other lineage markers (CD31, CD49a, CD90, and CD140b) in the 1390C1 hiPSC line. The $SIRPA^{+}/CD31^{-}/CD49a^{-}/CD90^{-}/CD140b^{-}$ population was gated as CMs. (b) Immunofluorescence analysis of MLC2v (green), MLC2a (red), and Hoechst (blue) in day-27 AIC- and VIC-CMs derived from the 1390C1 hiPSC line. Scale bars = 100 µm. (c) Histogram showing expression of MLC2a and 2v in AIC- and VIC-CMs on day 60. (d) Expression frequencies of all markers in $TNNI1^{+}$ CMs, analyzed using the Lyoplate Human Cell Surface Marker Screening Panel. (e) Flow cytometry histograms of CD57 (i), CD71 (ii), CD98 (iii), SSEA-4 (iv), and CD151 (v) with isotype control expressions in AIC-, AIC/woRA-, and VIC-CMs. (f) Representative flow cytometry images of day-20 AIC- and VIC-CMs stained with SSEA-4 in the TNNI1-EmGFP reporter hiPSC line.

Figure S2

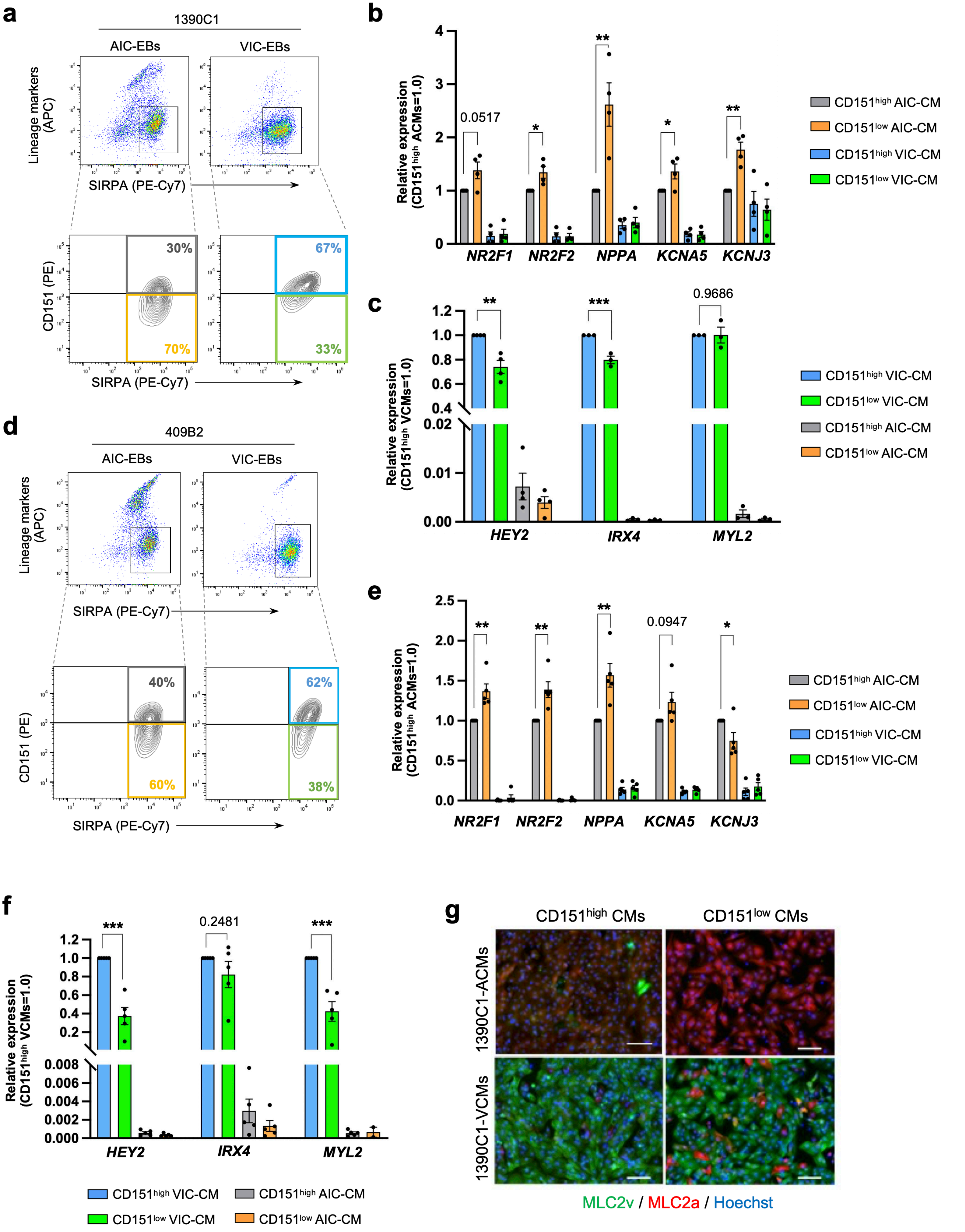

a
1390C1
AIC-EBs
VIC-EBs
Lineage markers (APC)
SIRPA (PE-Cy7)
CD151 (PE)
30%
70%
67%
33%
b
Relative expression (CD151high ACMs=1.0)
0.0517
NR2F1
NR2F2
NPPA
KCNA5
KCNJ3
CD151high AIC-CM
CD151low AIC-CM
CD151high VIC-CM
CD151low VIC-CM
c
Relative expression (CD151high VCMs=1.0)
0.9686
HEY2
IRX4
MYL2
d
409B2
AIC-EBs
VIC-EBs
40%
60%
62%
38%
e
0.0947
f
0.2481
g
CD151high CMs
CD151low CMs
1390C1-ACMs
1390C1-VCMs
MLC2v / MLC2a / Hoechst

Figure S2. Atrial- and ventricular-related gene expression in CMs derived from the 1390C1 and 409B2 hiPSC lines, related to Figure 1.

(a) Representative flow cytometry images of day 20 AIC- and VIC-EBs derived from the 1390C1 hiPSC line and stained with SIRPA, CD31, CD49a, CD90, CD140b, and CD151. The SIRPA$^+$/ CD31$^-$/CD49a$^-$/CD90$^-$/CD140b$^-$ population was gated as CMs (top), following which CD151 expression was analyzed in SIRPA$^+$/ CD31$^-$/CD49a$^-$ /CD90$^-$/CD140b$^-$ cells (bottom). (b) Relative expression of atrial-related genes in CD151^{low} AIC-CMs and CD151$^{high/low}$ VIC-CMs compared to those in CD151^{high} AIC-CMs derived from the 1390C1 hiPSC line. n=4 independent differentiation experiments per group. Data are expressed as mean ± SEM. Statistical analysis was conducted between CD151^{high} AIC-CM and CD151^{low} AIC-CM using the unpaired two-tailed t-test. * $p < 0.05$ and ** $p < 0.01$. (c) Relative expression of ventricular-related genes in CD151^{low} VIC-CMs and CD151$^{high/low}$ AIC-CMs compared to those in CD151^{high} VIC-CMs derived from the 1390C1 hiPSC line. n=3–4 independent differentiation experiments per group. Data are expressed as mean ± SEM. Statistical analysis was conducted between CD151^{high} VIC-CM and CD151^{low} VIC-CM using the unpaired two-tailed t-test. ** $p < 0.01$; *** $p < 0.001$. (d) Representative flow cytometry images of day-29 AIC- and VIC-EBs derived from the 409B2 hiPSC line and stained with SIRPA, CD31, CD49a, CD90, CD140b, and CD151. The SIRPA$^+$ population negative for the lineage markers (CD31, CD49a, CD90, and CD140b) was gated as CMs (top) and analyzed for CD151 expression (bottom). (e) Relative expression of atrial-related genes in CD151^{low} AIC-CMs and CD151$^{high/low}$ VIC-CMs compared to those in CD151^{high} AIC-CMs derived from the 409B2 hiPSC line. n=5 independent differentiation experiments per group. Data are expressed as mean ± SEM. Statistical analysis was conducted CD151^{high} AIC-CM and CD151^{low} AIC-CM using the unpaired two-tailed t-test. * $p <0.05$ and ** $p < 0.01$. (f) Relative expression of ventricular-related genes in CD151^{low} VIC-CMs and CD151$^{high/low}$ AIC-CMs compared to that in CD151^{high} VIC-CMs derived from the 409B2 hiPSC line. n=5 independent differentiation experiments per group. Data are expressed as mean ± SEM. Statistical analysis was conducted CD151^{high} VIC-CM and CD151^{low} VIC-CM using the unpaired two-tailed t-test. *** $p < 0.001$. (g) Representative immunofluorescence images of MLC2v (green), MLC2a (red), and Hoechst (blue) in CD151$^{high/low}$ AIC-CMs and in CD151$^{high/low}$ VIC-CMs on day 27. Scale bars = 100 µm.

Figure S3

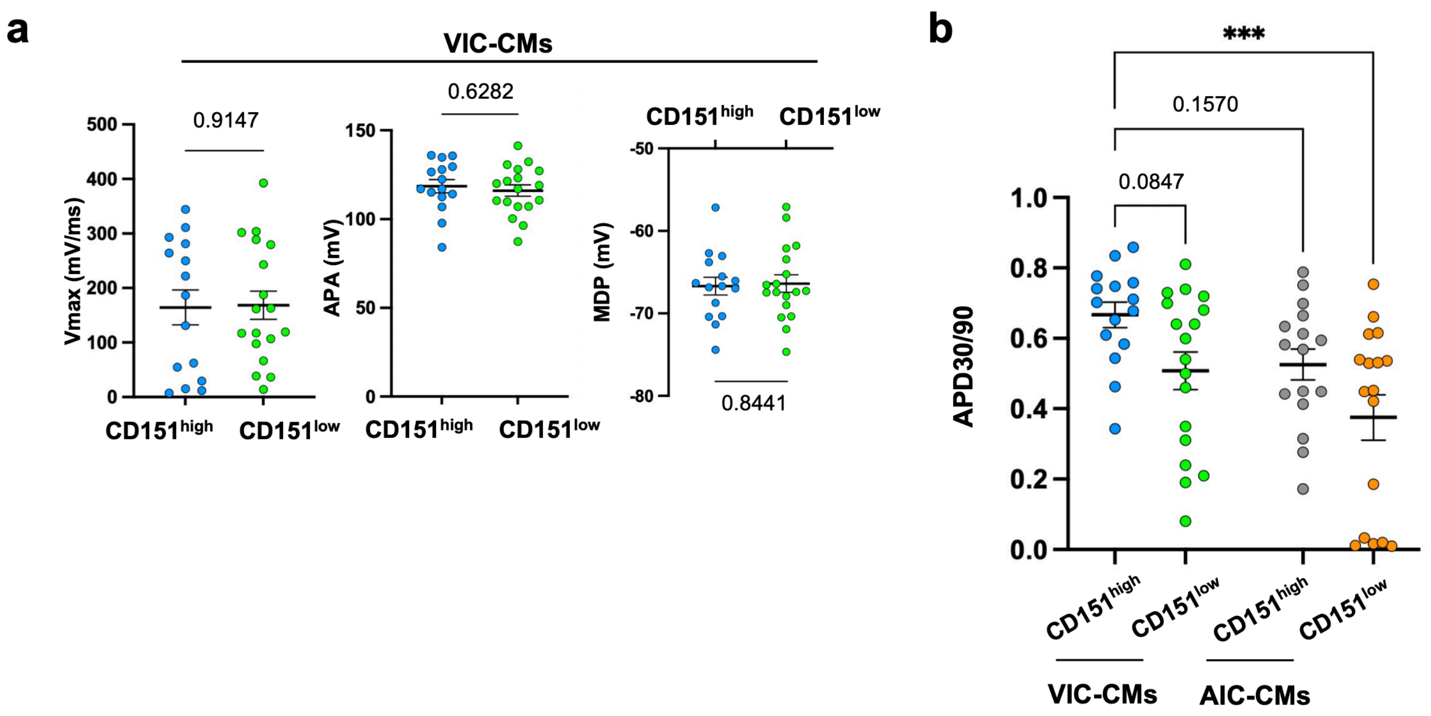

Figure S3. Electrophysiological properties in CD151high/low AIC-CMs and ViC-CMs, related to Figure 2.

(a) Electrophysiology measurements of Vmax (mV/ms), APA, and MDP in CD151high VCMs (n=15) and CD151low VCMs (n=18) from three independent differentiation experiments. Data are presented as mean ± SEM. Statistical analysis was conducted using an unpaired two-tailed t-test. (b) APD30/90 of CD151high VCMs (n=15), CD151low VCMs (n=18), CD151high ACMs (n=16), and CD151low ACMs (n=17). Data are presented as mean ± SEM. Statistical analysis was conducted using one-way ANOVA followed by Dunnett's test. *** $p < 0.001$.

Figure S4

a

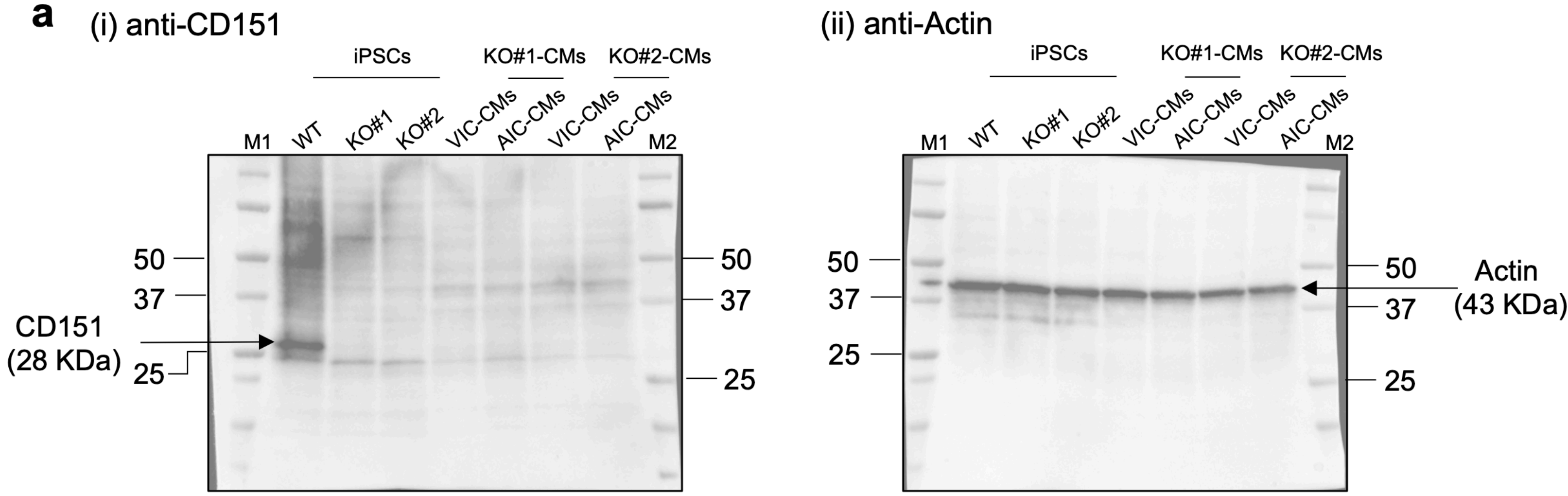

b

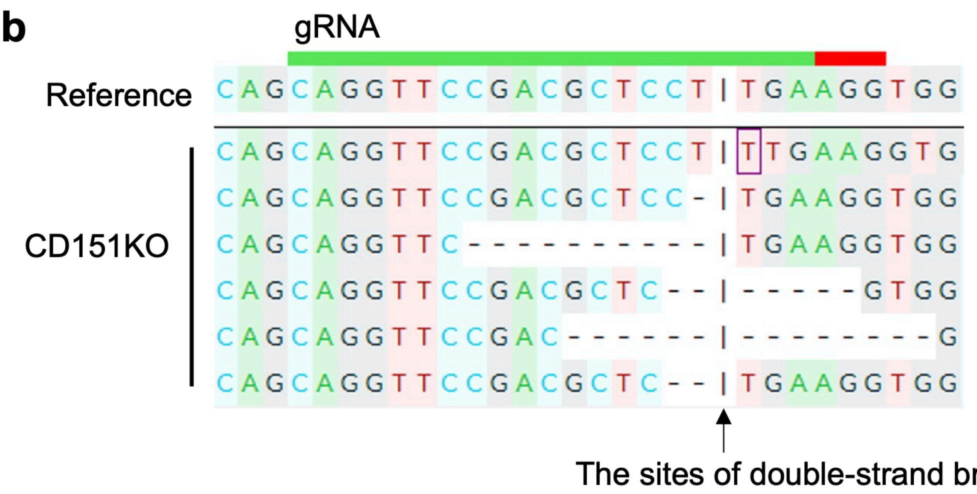

Indel (bp)	%
1	68.4
-1	14.5
-10	6.1
-7	4.3
-14	3.5
-2	3.2

c

(i) *FDXR*

WT: GTAACTGAATCATCTCCCGAAGCTCCTTGAAGGTGGGAGC
CD151KO: GTAACTGAATCATCTCCCGAAGCTCCTTGAAGGTGGGAGC

(ii) *MBTD1*

WT: ATAATTTCCTTCAAGGAGCGTCTGATCAGGAAAGCAATGG
CD151KO: ATAATTTCCTTCAAGGAGCGTCTGATCAGGAAAGCAATGG

(iii) *LYST*

WT: TCTTAGAGCCTTCAGGGAGAGTTGGAACCAGCATCATTTTCC
CD151KO: TCTTAGAGCCTTCAGGGAGAGTTGGAACCAGCATCATTTTCC

(iv) *SPINT2*

WT: GCCTCGCGTTCAGGTGCCGACGCTCCGGGAGGGTCCGCCA
CD151KO: GCCTCGCGTTCAGGTGCCGACGCTCCGGGAGGGTCCGCCA

(v) *C14orf166*

WT: TCGAGAGCCGTCAACTTGCGTCGGAACATGGTCCCCGCTT
CD151KO: TCGAGAGCCGTCAACTTGCGTCGGAACATGGTCCCCGCTT

d

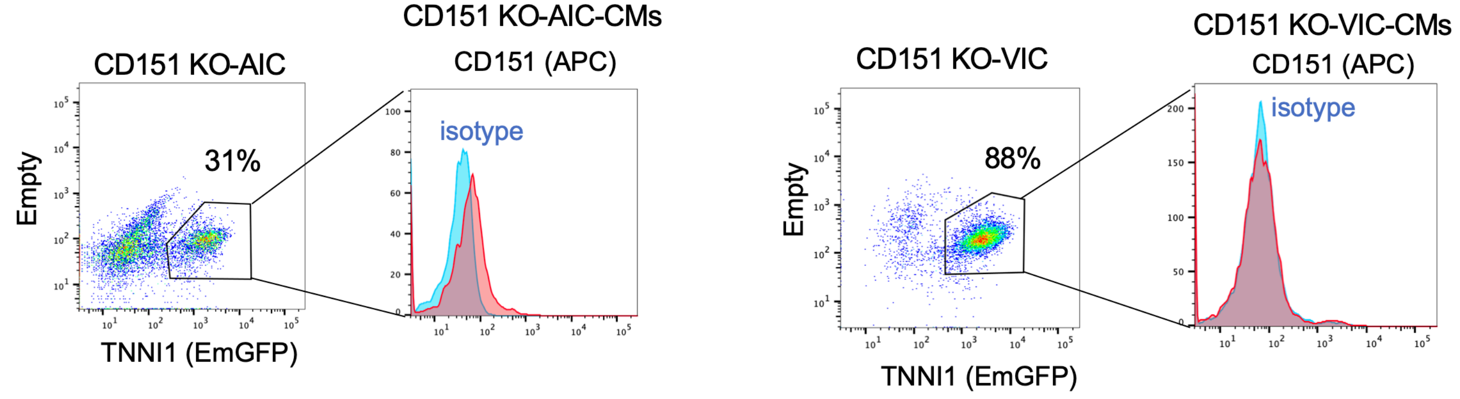

Figure S4. CD151 KO hiPSCs differentiation, related to Figure 3.

(a) Western blots image of CD151 expression (i) and Actin (ii) in WT-hiPSCs, CD151 KO hiPSCs, VIC-, and AIC-CMs. KO#1 hiPSCs were generated by different gRNA (5'-AACGAGAAGAAGACAACATG-3') than the one mentioned in the manuscript. Fig. 3b showed the result of KO#2 hiPSCs , and they were used for further experiments. M1 and M2 indicate different markers. (b) Sanger sequencing of a target site in the *CD151* gene. The gRNA and PAM sequences are indicated in green and red above the reference sequence, respectively. The right table shows the indel size (bp) and the population (%) of each indel observed in CD151KO hiPSCs. (c) DNA sequences of five predicted off-target genes. The black boxes indicate the off-target sequences of WT cells (TNNI1-EmGFP hiPSCs). (d) Representative flow cytometry images of day-20 AIC- (left) and VIC-EBs (right) derived from CD151 KO hiPSCs. The gating populations were TNNI1^{+} CMs. Histogram comparing the isotype control (blue) and CD151 staining (red) are shown for each differentiation.

Figure S5

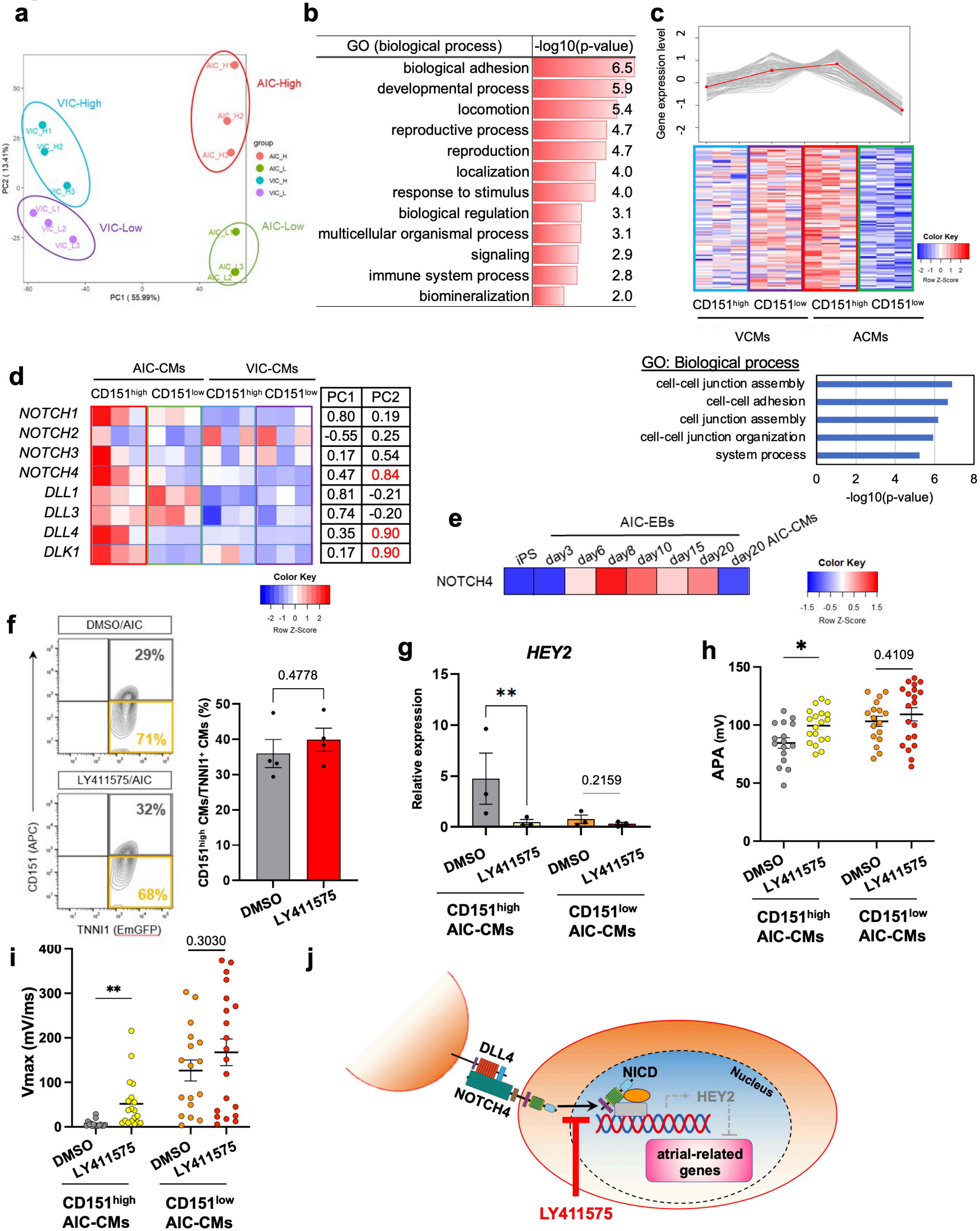

a
VIC-High
AIC-High
VIC-Low
AIC-Low
group
AIC_H
AIC_L
VIC_H
VIC_L
PC1 (55.99%)
PC2 (13.41%)
b
GO (biological process)
-log10(p-value)
biological adhesion 6.5
developmental process 5.9
locomotion 5.4
reproductive process 4.7
reproduction 4.7
localization 4.0
response to stimulus 4.0
biological regulation 3.1
multicellular organismal process 3.1
signaling 2.9
immune system process 2.8
biomineralization 2.0
c
Gene expression level
Color Key
Row Z-Score
CD151high CD151low CD151high CD151low
VCMs
ACMs
GO: Biological process
cell-cell junction assembly
cell-cell adhesion
cell junction assembly
cell-cell junction organization
system process
-log10(p-value)
d
AIC-CMs
VIC-CMs
CD151high CD151low CD151high CD151low
PC1 PC2
NOTCH1 0.80 0.19
NOTCH2 -0.55 0.25
NOTCH3 0.17 0.54
NOTCH4 0.47 0.84
DLL1 0.81 -0.21
DLL3 0.74 -0.20
DLL4 0.35 0.90
DLK1 0.17 0.90
Color Key
Row Z-Score
e
AIC-EBs
iPS day3 day6 day8 day10 day15 day20 day20 AIC-CMs
NOTCH4
Color Key
Row Z-Score
f
DMSO/AIC
29%
71%
LY411575/AIC
32%
68%
CD151 (APC)
TNNI1 (EmGFP)
CD151high CMs/TNNI1+ CMs (%)
0.4778
DMSO
LY411575
g
HEY2
Relative expression
**
0.2159
DMSO LY411575 DMSO LY411575
CD151high AIC-CMs
CD151low AIC-CMs
h
APA (mV)
*
0.4109
DMSO LY411575 DMSO LY411575
CD151high AIC-CMs
CD151low AIC-CMs
i
Vmax (mV/ms)
**
0.3030
DMSO LY411575 DMSO LY411575
CD151high AIC-CMs
CD151low AIC-CMs
j
DLL4
NOTCH4
NICD
Nucleus
HEY2
atrial-related genes
LY411575

Figure S5. Transcriptome analysis of CD151$^{high/low}$ CMs and Notch signaling inhibition in AIC differentiation, related to Figure 4.

(a) PCA showing the correlation between CD151 expression and differentiation conditions. n=3 independent experiments per group. (b) GO enrichment analysis of 186 DEGs with factor loadings of PC2 < -0.8 or > 0.8 in AIC-CMs. Twelve biological processes are shown with a significance level of $p < 0.05$. (c) This group consisted of 88 clustered genes that showed a lower expression in CD151^{low} AIC-CMs via hierarchical clustering of CD151$^{high/low}$ VIC-CMs and CD151$^{high/low}$ AIC-CMs. Expression patterns of each gene (grey) and the average of all 88 genes are shown above. GO enrichment (biological process) is shown below. (d) Heatmap of the expression changes of Notch-related genes in CD151$^{high/low}$ AIC- and VIC-CMs. The PC1 and PC2 factor loadings for each gene are shown in the table (right). (e) Heatmap of the *NOTCH4* mRNA time-course expression during AIC differentiation. (f) Representative flow cytometry images of TNNI1+ CMs differentiated with DMSO (top) or LY411575 (bottom) and stained with CD151 antibody. The percentages represent gated populations. The bar graph indicates the percentage of CD151^{high} AIC-CMs gated from TNNI1^{+} CMs differentiated with DMSO or LY411575. n=4 independent differentiation experiments per group. Data are expressed as mean ± SEM. Statistical analysis compared the two groups using an unpaired two-tailed t-test. (g) Relative expression of *HEY2* in CD151$^{high/low}$ AIC-CMs differentiated with LY411575 or DMSO as vehicle control. n=3 independent differentiation experiments per group. Data are expressed as mean ± SEM. Statistical analysis was conducted using the paired two-tailed t-test. ** $p < 0.01$. (h) Electrophysiological measurements of APA in CD151$^{high/low}$ AIC-CMs differentiated with vehicle treatment (DMSO) or LY411575. CD151^{high} AIC-CMs, DMSO (n=16) and LY411575 treatment (n=19); CD151^{low} AIC-CMs, vehicle (DMSO) (n=17) and LY411575 treatment (n=20). Data are presented as mean ± SEM. Statistical analysis was performed using the unpaired two-tailed t-test. * $p < 0.05$. (i) Electrophysiological measurements of Vmax (mV/ms) in CD151$^{high/low}$ AIC-CMs differentiated with vehicle treatment (DMSO) or LY411575. CD151^{high} AIC-CMs, DMSO (n=16) and LY411575 treatment (n=19); CD151^{low} AIC-CMs, vehicle (DMSO) (n=17) and LY411575 treatment (n=20). Data are presented as mean ± SEM. Statistical analysis was performed using an unpaired two-tailed t-test ** $p < 0.01$. (j) A schematic diagram of the putative atrial gene upregulation mechanism induced by Notch inhibition (LY411575). Notch signaling inhibits atrial-related gene expression by inducing *HEY2* transcription as a result of Notch intercellular domain (NICD) binding to the transcriptional activation complex in the *HEY2* promoter region. LY411575 inhibits Notch cleavage and NICD generation, thus derepressing the inhibition of atrial-related genes to induce atrial differentiation.

Figure S6

a

GO (biological process)	-log10(p value)
reproductive process	5.4
reproduction	5.3
locomotion	3.3
developmental process	3.2
biological adhesion	2.7
biological regulation	2.5
rhythmic process	2.5
response to stimulus	2.1
cellular process	2.0
growth	1.9
multicellular organismal process	1.7
multi-organism process	1.6

b

Reactome pathway	-log10(p value)
Cell Cycle	10.3
Mitotic G1 phase and G1/S transition	9.8
Cell Cycle, Mitotic	9.6
DNA Repair	6.2
S Phase	5.1
Cell Cycle Checkpoints	3.0
Homology Directed Repair	2.9
Transcriptional regulation by the AP-2 (TFAP2) family of transcription factors	2.9
DNA Double-Strand Break Repair	2.9
Binding and Uptake of Ligands by Scavenger Receptors	2.5
Synthesis of DNA	2.5
Signaling by NOTCH	2.2
Meiotic recombination	2.2
Mitotic G2-G2/M phases	2.1
HDR through Homologous Recombination (HRR) or Single Strand Annealing (SSA)	2.1
Fanconi Anemia Pathway	2.0

c

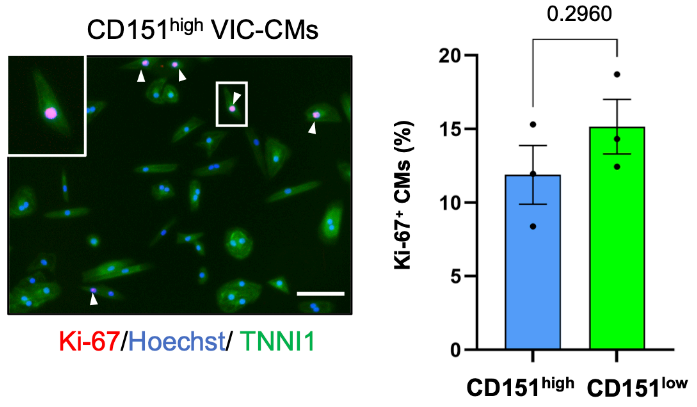

d

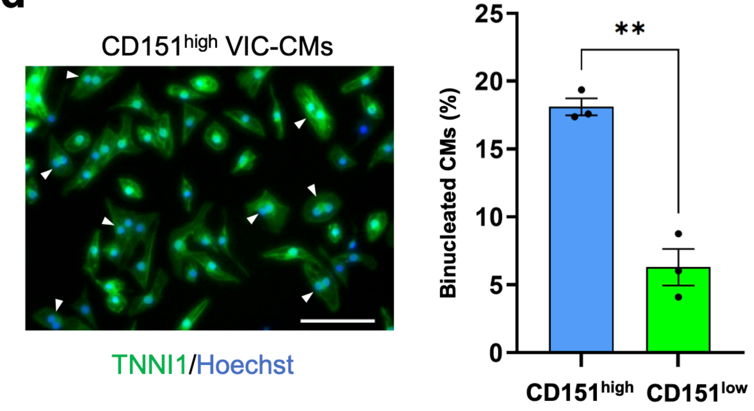

Figure S6. Transcriptome analysis of CD151$^{high/low}$ VIC-CM, related to Figure 5.

(a) GO enrichment analysis of DEGs between CD151^{high} and CD151^{low} VIC-CMs. Twelve biological processes are shown with a significance level of $p < 0.05$. (b) Reactome pathway analysis of DEGs between CD151$^{high/low}$ VIC-CMs. Sixteen biological processes are shown with a significance level of $p < 0.05$. (c) Representative immunofluorescence images of Ki-67 (red), Hoechst (blue), and TNNI1 (green) in CD151^{high} VIC-CMs derived from the TNNI1-EmGFP reporter hiPSC line. Ki-67^{+} CMs are indicated by the arrowheads. The inset is a high magnification image of the boxed area and shows Ki-67^{+} CMs. Scale bar = 100 μm. Bar graph shows percentage of Ki-67^{+} CMs in CD151^{high} VIC-CMs (n=1,461) and CD151^{low} VIC-CMs (n=1,077) derived from the TNNI1-EmGFP reporter hiPSC line. n=30 images from three independent differentiation experiments. Data are expressed as mean ± SEM of three experiments. Statistical analysis was conducted using an unpaired two-tailed t-test. (d) Representative immunofluorescence images of TNNI1 (green) and Hoechst (blue) in CD151^{high} VIC-CMs derived from the TNNI1-EmGFP reporter hiPSC line. Binuclear CMs are indicated by the arrowheads. Scale bar = 100 μm. Bar graph shows percentage of binuclear CMs in CD151^{high} VIC-CMs (n=1,236) and CD151^{low} VIC-CMs (n=720) derived from the TNNI1-EmGFP reporter hiPSC line. n=15 images from three independent differentiation experiments. Data are expressed as mean ± SEM of three experiments. Statistical analysis was conducted using an unpaired two-tailed t-test. ** $p < 0.01$.

Figure S7

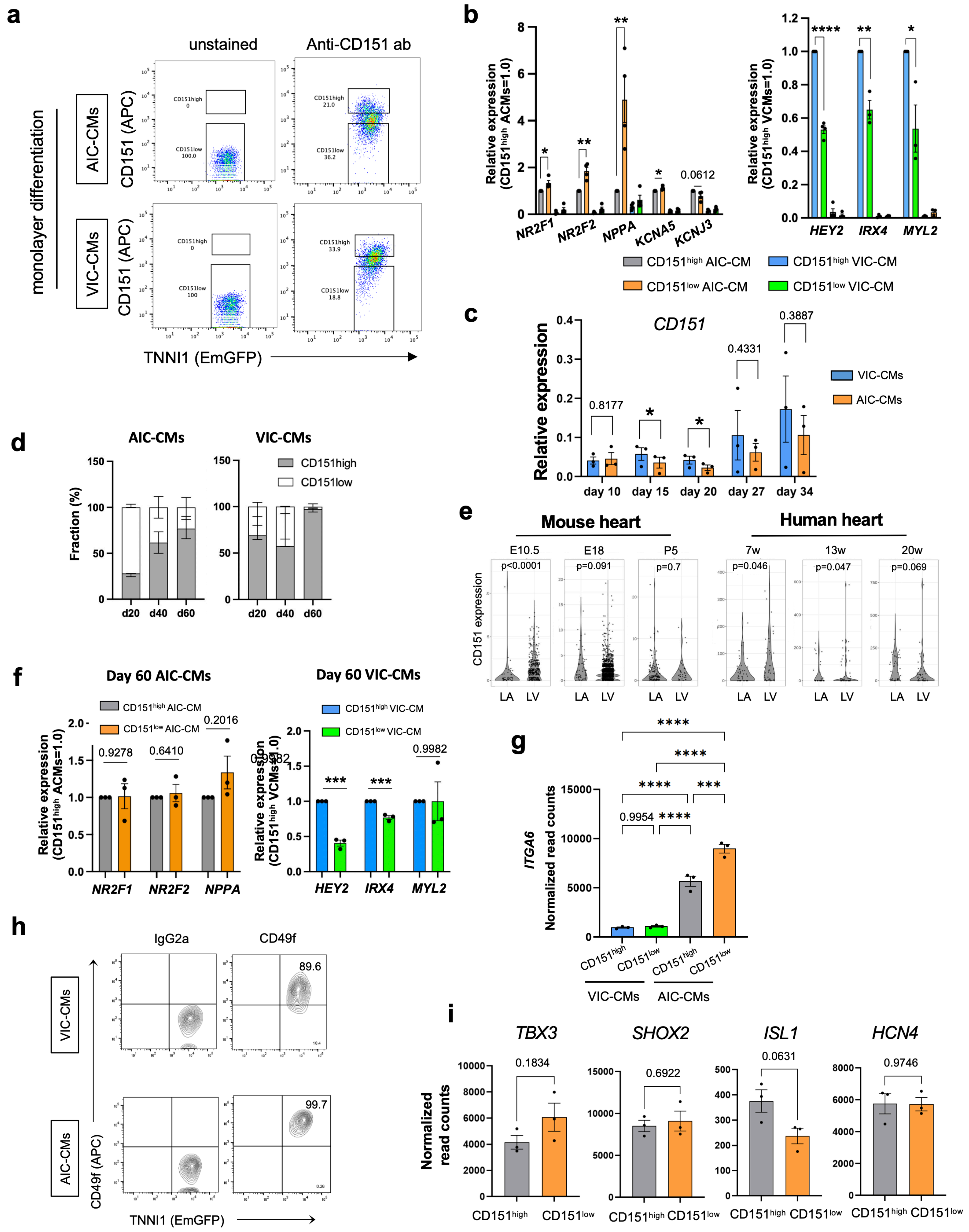

a
unstained
Anti-CD151 ab
monolayer differentiation
AIC-CMs
VIC-CMs
CD151 (APC)
CD151high 0
CD151low 100.0
CD151high 21.0
CD151low 36.2
CD151high 0
CD151low 100
CD151high 33.9
CD151low 18.8
TNNI1 (EmGFP)
b
Relative expression (CD151high ACMs=1.0)
NR2F1
NR2F2
NPPA
KCNA5
KCNJ3
0.0612
Relative expression (CD151high VCMs=1.0)
HEY2
IRX4
MYL2
CD151high AIC-CM
CD151low AIC-CM
CD151high VIC-CM
CD151low VIC-CM
c
CD151
Relative expression
0.8177
0.4331
0.3887
day 10
day 15
day 20
day 27
day 34
VIC-CMs
AIC-CMs
d
AIC-CMs
VIC-CMs
Fraction (%)
CD151high
CD151low
d20
d40
d60
e
Mouse heart
Human heart
E10.5
E18
P5
7w
13w
20w
p<0.0001
p=0.091
p=0.7
p=0.046
p=0.047
p=0.069
CD151 expression
LA
LV
f
Day 60 AIC-CMs
Day 60 VIC-CMs
CD151high AIC-CM
CD151low AIC-CM
CD151high VIC-CM
CD151low VIC-CM
Relative expression (CD151high ACMs=1.0)
Relative expression (CD151high VCMs=1.0)
0.9278
0.6410
0.2016
0.9982
NR2F1
NR2F2
NPPA
HEY2
IRX4
MYL2
g
ITGA6
Normalized read counts
0.9954
CD151high
CD151low
VIC-CMs
AIC-CMs
h
IgG2a
CD49f
89.6
99.7
VIC-CMs
AIC-CMs
CD49f (APC)
TNNI1 (EmGFP)
i
TBX3
SHOX2
ISL1
HCN4
Normalized read counts
0.1834
0.6922
0.0631
0.9746
CD151high
CD151low

Figure S7. CD151 selection in monolayer culture, CD151 expression over time in vitro culture and in vivo heart tissues, *ITGA6* and CD49f expression in VIC- and AIC-CMs, and sinoatrial marker genes expression in AIC-CMs.

(a) Representative flow cytometry images of day-20 AIC- and VIC-CMs with monolayer differentiation stained with the CD151 antibody. (b) Relative expression of atrial-related genes in $CD151^{low}$ ACMs and $CD151^{high/low}$ VCMs compared to those in $CD151^{high}$ ACMs derived from the TNNI1-EmGFP hiPSC line with monolayer differentiation (top). Relative expression of ventricular-related genes in $CD151^{low}$ VCMs and $CD151^{high/low}$ ACMs compared to those in $CD151^{high}$ VCMs derived from the TNNI1-EmGFP hiPSC line with monolayer differentiation (bottom). n=3 independent experiments per group. Data are expressed as mean ± SEM. Statistical analysis was conducted using the unpaired two-tailed t-test. * $p < 0.05$, ** $p < 0.01$; *** $p < 0.001$, and **** $p < 0.0001$. (c) Time-course of relative *CD151* expression. n=3 independent differentiation experiments per group. Data are expressed as mean ± SEM. Statistical analysis was conducted using a paired two-tailed t-test. * $p < 0.05$. (d) The fraction of $CD151^{high}$ and $CD151^{low}$ population at day 20, day 40, and day 60 after differentiation. Data are shown as median with 95% CI. (e) *CD151* expression in the left atrium (LA) and ventricle (LV) of mouse and human across various stages derived from single-cell RNA sequence data. (f) Atrial marker genes expression (top) and ventricular genes expression (bottom) on day-60 $CD151^{high/low}$ ACMs and VCMs sorted on day 20. n=3 independent differentiation experiments per group. Statistical analysis was conducted using an unpaired two-tailed t-test. *** $p < 0.001$. (g) *ITGA6* gene expression in $CD151^{high}$ VCMs and $CD151^{high/low}$ ACMs. Statistical analysis was conducted using one-way ANOVA followed by Tukey's HSD test. *** $p < 0.001$ and **** $p < 0.0001$. (h) Flow cytometry images of VIC- and AIC-CMs stained with isotype control (left) and CD49f antibodies. The percentage of positive cells of CD49f in $TNNI1^{+}$ CMs represents the gated populations. (i) Sinoatrial marker gene expressions in $CD151^{high/low}$ ACMs. n=3 independent differentiation experiments per group. Statistical analysis was conducted using an unpaired two-tailed t-test.

Figure S8

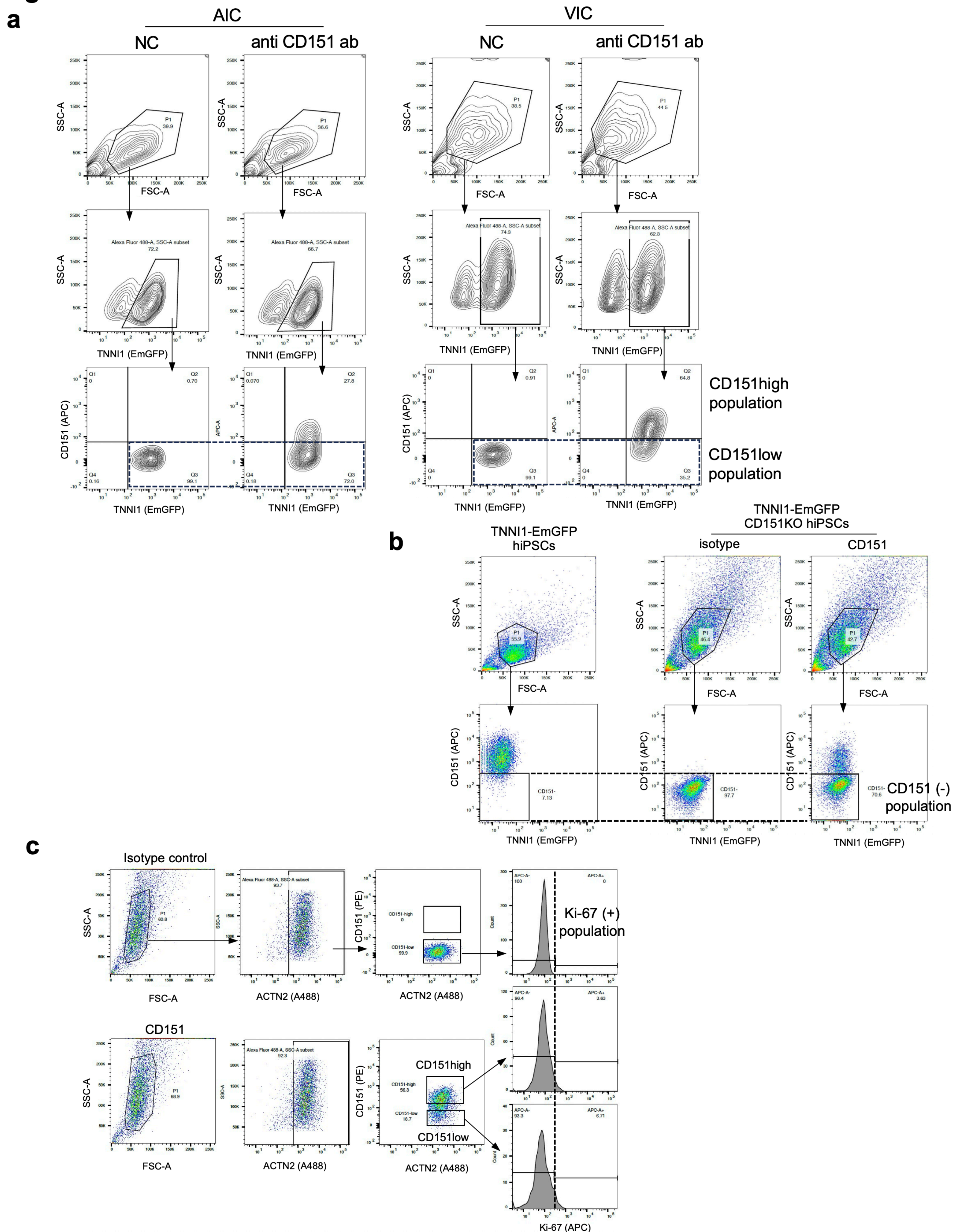
a
AIC
VIC
NC
anti CD151 ab
SSC-A
FSC-A
P1
39.9
36.6
38.5
44.5
Alexa Fluor 488-A, SSC-A subset
72.2
66.7
74.3
62.3
TNNI1 (EmGFP)
CD151 (APC)
APC-A
Q1
Q2
Q3
Q4
CD151high population
CD151low population
b
TNNI1-EmGFP hiPSCs
TNNI1-EmGFP CD151KO hiPSCs
isotype
CD151
P1
55.9
46.4
42.7
CD151-
7.13
97.7
70.6
CD151 (-) population
c
Isotype control
CD151
P1
60.8
68.9
Alexa Fluor 488-A, SSC-A subset
93.7
92.3
ACTN2 (A488)
CD151 (PE)
CD151-high
CD151-low
CD151high
CD151low
APC-A-
APC-A+
Ki-67 (+) population
Count
Ki-67 (APC)

Figure S8. Gating strategy for flow cytometry analysis.

(a) Gating strategy for CD151$^{high/low}$ CMs derived from TNNI1-EmGFP hiPSCs. TNNI1-positive cells were gated as cardiomyocytes and separated by CD151 expression levels. CD151 low expressed population was determined by Negative control (NC). (b) CD151KO hiPSCs were generated by sorting the CD151-negative population as determined with the isotype control sample population. (c) 1390C1-derived CMs were gated as an ACTN2-positive population. Those CMs were categorized based on CD151 expression, and Ki-67 expressing population was analyzed in each CD151^{high} and CD151^{low} CMs.

Supplementary Table 1. APD30/90 in CD151$^{high/low}$ AIC-CMs and VIC-CMs.

	AIC-CMs (APD30/90)		
	CD151^{high}		CD151^{low}
1	0.44	1	0.53
2	0.28	2	0.75
3	0.45	3	0.61
4	0.66	4	0.45
5	0.45	5	0.66
6	0.17	6	0.54
7	0.41	7	0.19*
8	0.31	8	0.62
9	0.58	9	0.42
10	0.59	10	0.02*
11	0.79	11	0.54
12	0.57	12	0.45
13	0.75	13	0.01*
14	0.70	14	0.02*
15	0.63	15	0.01*
16	0.61	16	0.53
		17	0.03*

	VIC-CMs (APD30/90)		
no.	CD151high	no.	CD151low
1	0.78	1	0.08*
2	0.86	2	0.64
3	0.76	3	0.81
4	0.61	4	0.54
5	0.65	5	0.73
6	0.58	6	0.19*
7	0.71	7	0.6
8	0.54	8	0.46
9	0.68	9	0.68
10	0.74	10	0.74
11	0.34	11	0.72
12	0.75	12	0.31
13	0.83	13	0.21*
14	0.7	14	0.5
15	0.46	15	0.64
		16	0.7
		17	0.24*
		18	0.35

*APD30/90 < 0.3 and Vmax > 10 indicates atrial properties of APs.

Supplementary Table 2. List of Taqman probes used in this study.

Target gene	Assay ID
NR2F1	Hs00818842_m1
NR2F2	Hs00819630_m1
NPPA	Hs00383230_g1
KCNA5	Hs00969279_s1
KCNJ3	Hs00158421_m1
HEY1	Hs01114113_m1
HEY2	Hs01012057_m1
IRX4	Hs00212560_m1
MYL2	Hs00166405_m1
NOTCH4	Hs00965889_m1
DLL4	Hs00184092_m1
TBX5	Hs00361155_m1
MYL7	Hs01085598_g1
GAPDH	Hs99999905_m1

Supplementary Table 3. List of primers used for Sanger sequencing.

Target	Sequences (5' to 3')
CD151_F	ACCCTCCCTGAAGCTTTCTT
CD151_R	CAGATGCGATGACCTTTGTG
FDXR_F	GATGGAAGAAGGGGAGCAGT
FDXR_R	GGACCTCTGTCAGCAACGTA
MBTD1_F	TGTAAAGCTGACGGAACACG
MBTD1_R	CCCACCTGAAAAATCTGGAA
LYST_F	TTTGAGGTGTTTTGCATTGG
LYST_R	GTGGCCCATGAGCACTTAAA
SPINT2_F	CGTGGATGCTGCGTTCTC
SPINT2_R	GGGAGCCGCTTCCAATAG
C14orf166_F	GCTTTAAAGGCGGGAAGG
C14orf166_R	ACCGCTTTTAAGCCACGATT
