# Supplementary material for: CD151 expression marks atrial- and ventricular- differentiation from human induced pluripotent stem cells
Source: Commun Biol. 2024 Feb 28;7:231. doi: 10.1038/s42003-024-05809-2 (PMC10901864; doi:10.1038/s42003-024-05809-2)
Supplement: Supplementary file 6 — Reporting Summary [file 42003_2024_5809_MOESM6_ESM.pdf]

Corresponding author(s): Yoshinori Yoshida and Kenji Miki
Last updated by author(s): 2023/12/30

Reporting Summary

Nature Portfolio wishes to improve the reproducibility of the work that we publish. This form provides structure for consistency and transparency in reporting. For further information on Nature Portfolio policies, see our Editorial Policies and the Editorial Policy Checklist.

Statistics

For all statistical analyses, confirm that the following items are present in the figure legend, table legend, main text, or Methods section.

n/a	Confirmed	
☐	☒	The exact sample size (*n*) for each experimental group/condition, given as a discrete number and unit of measurement
☐	☒	A statement on whether measurements were taken from distinct samples or whether the same sample was measured repeatedly
☐	☒	The statistical test(s) used AND whether they are one- or two-sided<br>*Only common tests should be described solely by name; describe more complex techniques in the Methods section.*
☒	☐	A description of all covariates tested
☐	☒	A description of any assumptions or corrections, such as tests of normality and adjustment for multiple comparisons
☐	☒	A full description of the statistical parameters including central tendency (e.g. means) or other basic estimates (e.g. regression coefficient) AND variation (e.g. standard deviation) or associated estimates of uncertainty (e.g. confidence intervals)
☐	☒	For null hypothesis testing, the test statistic (e.g. *F*, *t*, *r*) with confidence intervals, effect sizes, degrees of freedom and *P* value noted<br>*Give P values as exact values whenever suitable.*
☒	☐	For Bayesian analysis, information on the choice of priors and Markov chain Monte Carlo settings
☒	☐	For hierarchical and complex designs, identification of the appropriate level for tests and full reporting of outcomes
☒	☐	Estimates of effect sizes (e.g. Cohen's *d*, Pearson's *r*), indicating how they were calculated

Our web collection on statistics for biologists contains articles on many of the points above.

Software and code

Policy information about availability of computer code

Data collection	RNA-seq: llumina Nextseq 500<br>Fluoresence Microscopy: BZ-X700 (Keyence)<br>Flow Cytometry: BD FACSAria Fusion (BD), FACSLyric (BD)<br>Patch clamp: Axopatch 200B amplifier (Molecular Devices), DigiData (1322A) and pCLAMP(v9.2)<br>Optical recording of action potentials: AQUACOSMOS 2.6 (Hamamatsu Photonics)
Data analysis	RNA-seq: R (v4.0.3), R package gplots (v3.0.1), STAR (v2.7.3a),<br>Flow cytometric analysis: BD FACSDiva (v.8.0.1), BD FACSuite (v.1.2.1), and FlowJo (v10.7.1, v10.9.0)<br>Imaging: ImageJ (v1.50e)<br>Patch clamp: pCLAMP(v10.7)<br>Optical recording of action potentials: OriginPro 2021, 2023b (OriginLab)<br>Statistical analysis: GraphPad Prism (v9.0.1)<br>Gene editng efficiency analysis: DECODR v3.0 (https://decodr.org/)

For manuscripts utilizing custom algorithms or software that are central to the research but not yet described in published literature, software must be made available to editors and reviewers. We strongly encourage code deposition in a community repository (e.g. GitHub). See the Nature Portfolio guidelines for submitting code & software for further information.

Data

Policy information about availability of data

All manuscripts must include a data availability statement. This statement should provide the following information, where applicable:

- Accession codes, unique identifiers, or web links for publicly available datasets
- A description of any restrictions on data availability
- For clinical datasets or third party data, please ensure that the statement adheres to our policy

The RNA-seq data reported in this paper have been deposited in NCBI's Gene Expression Omnibus and are accessible through GEO series accession number GSE179769. The data that support the findings of this study are available from the corresponding author upon reasonable request.

Human research participants

Policy information about studies involving human research participants and Sex and Gender in Research.

Reporting on sex and gender	*Use the terms sex (biological attribute) and gender (shaped by social and cultural circumstances) carefully in order to avoid confusing both terms. Indicate if findings apply to only one sex or gender; describe whether sex and gender were considered in study design whether sex and/or gender was determined based on self-reporting or assigned and methods used. Provide in the source data disaggregated sex and gender data where this information has been collected, and consent has been obtained for sharing of individual-level data; provide overall numbers in this Reporting Summary. Please state if this information has not been collected. Report sex- and gender-based analyses where performed, justify reasons for lack of sex- and gender-based analysis.*
Population characteristics	*Describe the covariate-relevant population characteristics of the human research participants (e.g. age, genotypic information, past and current diagnosis and treatment categories). If you filled out the behavioural & social sciences study design questions and have nothing to add here, write "See above."*
Recruitment	*Describe how participants were recruited. Outline any potential self-selection bias or other biases that may be present and how these are likely to impact results.*
Ethics oversight	*Identify the organization(s) that approved the study protocol.*

Note that full information on the approval of the study protocol must also be provided in the manuscript.

Field-specific reporting

Please select the one below that is the best fit for your research. If you are not sure, read the appropriate sections before making your selection.

☒ Life sciences ☐ Behavioural & social sciences ☐ Ecological, evolutionary & environmental sciences

For a reference copy of the document with all sections, see nature.com/documents/nr-reporting-summary-flat.pdf

Life sciences study design

All studies must disclose on these points even when the disclosure is negative.

Sample size	Required experimental sample sizes were estimated based on previous established protocols in the field (e.g. Kratsios, P. et al., Cell Stem Cell 2017). The sample sizes were adequate as the differences between experimental groups were reproducible. All n values are clearly indicated within the figure legends.
Data exclusions	No data was excluded for the analyses.
Replication	All experiments were repeated multiple times as indicated in each figure legend.
Randomization	Randomization is not applicable to this study because we did not perform any experiments where there are treatment and control groups that would necessitate randomization between the subjects.
Blinding	Blinding is not applicable to this study because we did not perform any experiments where there are treatment and control groups that would necessitate blinding.

Reporting for specific materials, systems and methods

We require information from authors about some types of materials, experimental systems and methods used in many studies. Here, indicate whether each material, system or method listed is relevant to your study. If you are not sure if a list item applies to your research, read the appropriate section before selecting a response.

Materials & experimental systems

n/a	Involved in the study
☐	☒ Antibodies
☐	☒ Eukaryotic cell lines
☒	☐ Palaeontology and archaeology
☒	☐ Animals and other organisms
☒	☐ Clinical data
☒	☐ Dual use research of concern

Methods

n/a	Involved in the study
☒	☐ ChIP-seq
☐	☒ Flow cytometry
☒	☐ MRI-based neuroimaging

Antibodies

Antibodies used

Mouse monoclonal anti-human CD151 (BD Biosciences, 556056, Clone 14A2.H1, dilutioin 1:200)
Mouse monoclonal PE anti-human CD151 antibody (BD Biosciences, 556057, Clone 14A2.H1, dilutioin 1:200)
Mouse monoclonal PE/Cyanine7 anti-human CD172a/b (SIRPα/β) antibody (BioLegend, 323808, Clone SE5A5, dilution 1:500)
Mouse monoclonal APC anti-human CD90 antibody (BD Biosciences, 559869, Clone 5E10, dilution 1:2500)
Mouse monoclonal APC anti-human CD31 antibody (BioLegend, 303116, Clone WM59, dilution 1:500)
Mouse monoclonal Alexa Fluor® 647 anti-human CD49a antibody (BioLegend, 328310, Clone TS2/7, dilution 1:500)
Mouse monoclonal APC anti-human CD140b antibody (BioLegend, 323608, Clone 18A2, dilution 1:500, dilution 1:500)
Rat monoclonal Alexa Fluor® 647 anti-mouse/human Ki-67 antibody (BioLegend, 151206, Clone 11F6, dilution 1:400)
Rabbit monoclonal anti-human ACTN2 antibody (Creative Diagnostics, DCABH-9438, Clone FQ3630Z, dilution 1:200)
Mouse monoclonal anti-MLC-2A antibody (Synaptic Systems, 311011, clone 56F5, dilution 1:100)
Rabbit polyclonal anti-MLC-2V antibody (Proteintech, 10906-1-AP, dilution 1:200)
APC anti-mouse IgG1 (BD Pharmingen, 560747(Component of Human Cell Surface Marker Screening Panel), dilution 1:200)
Alexa Fluor 647 goat anti-mouse IgG (H+L) (Thermo Fisher Scientific, A21236, dilution 1:500)
Alexa Fluor 647 goat anti-mouse IgG (H+L) (Thermo Fisher Scientific, A21235, dilution 1:500)
Alexa Fluor 647 goat anti-rabbit IgG (H+L) (Thermo Fisher Scientific, A21245, dilution 1:500)
Alexa Fluor 488 goat anti-rabbit IgG (H+L) (Thermo Fisher Scientific, A11034, dilution 1:500)
Alexa Fluor 488 donkey anti-rabbit IgG (H+L) (Thermo Fisher Scientific, A21206, dilution 1:500)
CD151 Monoclonal Antibody (2A8G8) (Thermo Fisher Scientific, 66567-1-IG, 1:2000)
Anti-Actin monoclonal antibody, clone C4(Merck Millipore, MAB1501, 1:5000)

Validation

Mouse monoclonal anti-human CD151: validated by the manufacture.
Mouse monoclonal PE anti-human CD151 antibody: validated by the manufacture.
Mouse monoclonal PE/Cyanine7 anti-human CD172a/b (SIRPα/β) antibody: Dubois, N.C. et al. Cell Stem Cell 2011
Mouse monoclonal APC anti-human CD90 antibody: Dubois, N.C. et al. Cell Stem Cell 2011
Mouse monoclonal APC anti-human CD31 antibody: Dubois, N.C. et al. Cell Stem Cell 2011
Mouse monoclonal Alexa Fluor® 647 anti-human CD49a antibody: Dubois, N.C. et al. Cell Stem Cell 2011
Mouse monoclonal APC anti-human CD140b antibody: Dubois, N.C. et al. Cell Stem Cell 2011
Rat monoclonal Alexa Fluor® 647 anti-mouse/human Ki-67 antibody: Kobayashi T. et al., Cell 2019
Rabbit monoclonal anti-human ACTN2 antibody: validated by the manufacture.
Mouse monoclonal anti-MLC-2A antibody: Burridge P.W. et al., Nature methods 2014
Rabbit polyclonal anti-MLC-2V antibody: Burridge P.W. et al., Nature methods 2014

Eukaryotic cell lines

Policy information about cell lines and Sex and Gender in Research

Cell line source(s)

The 1390C1 and 409B2 hiPSC lines were generated in CiRA. The TNNI1-EmGFP-reporter hiPSC line was generated in T-CiRA (Miki K et al., Nature Communications 2021).

Authentication

All hiPSC used in this study including reporter lines were validated with karyotype testing.

Mycoplasma contamination

All cell lines are regularly tested and were always negative for mycoplasma

Commonly misidentified lines
(See ICLAC register)

No commonly misidentified lines were used in this study.

Flow Cytometry

Plots

Confirm that:

☒ The axis labels state the marker and fluorochrome used (e.g. CD4-FITC).

☒ The axis scales are clearly visible. Include numbers along axes only for bottom left plot of group (a 'group' is an analysis of identical markers).

☒ All plots are contour plots with outliers or pseudocolor plots.

☒ A numerical value for number of cells or percentage (with statistics) is provided.

Methodology

Sample preparation	For Cell surface marker screening, EBs were dissociated into single cells by Liberase and Accumax. The dissociated cells were suspended with the antibodies of the Human Cell Surface Marker Screening Panel (BD Biosciences). For sorting of CMs derived from TNNI1-reporter hiPSC, EBs were dissociated into single cells by Liberase and Accumax. The dissociated cells were suspended in 2% FBS/PBS with purified mouse anti-human CD151 antibody (BD Biosciences, 1:200), and then stained by Alexa Fluor 647 goat anti-mouse IgG (Thermo Fisher Scientific, 1:200). For sorting of CMs derived from 1390C1 and 409B2 hiPSC lines, dissociated cells were stained with PE anti-human CD151 (BD Biosciences, 1:200), PE/Cyanine7 anti-human CD172a/b (SIRPα/β) Antibody (BioLegend, 1:500), APC Mouse Anti-Human CD90 (BD, 559869, Clone 5E10, 1:2500), APC anti-human CD31 Antibody (BioLegend, 1:500), Alexa Fluor® 647 anti-human CD49a Antibody (BioLegend, 1:500) and APC anti-mouse CD140b Antibody (BioLegend, 1:500). For Ki-67 analysis, the dissociated cells were fixed by 4% PFA, and then permeabilized with 1xPerm/Wash Buffer (BD Biosciences). Then the cells were stained with Alexa Fluor 647 anti-mouse/human Ki-67 antibody (BioLegend, 1:400) and PE anti-human CD151 (BD Biosciences, 1:200) and Alexa Fluor 488 donkey anti-rabbit IgG (Thermo Fisher Scientific, 1:200). For the binucleation analysis, the dissociated cells were stained with anti-human ACTN2 antibody (Creative Diagnostics, 1:200), Alexa Fluor 488 donkey anti-rabbit IgG (Thermo Fisher Scientific, 1:200), and Hoechst (DOJINDO, 1:1000).
Instrument	FACSAria Fusion, FACSLyric
Software	Acquisition: BD FACSDiva (v.8.0.1), BD FACSuite (v.1.0)<br>Analysis : Flowjo (v10.7.1, v10.9.0)
Cell population abundance	Flow cytometry analysis populations were generally >5%.
Gating strategy	Cells were gated on FSC/SSC first and an unstained sample as controls to establish the gate for positive cells. For screening, cells were gated on the isotype stained sample as control to establish the gate for positive cells. For sorting of CMs, cells were gated on an unstained sample as control to establish the gate for positive cells. For Ki-67 staining cells were gated on an unstained sample as control to establish the gate for positive cells.

☒ Tick this box to confirm that a figure exemplifying the gating strategy is provided in the Supplementary Information.
